# The Big Picture: 26 Years of Longitudinal Findings About Health Promotion and Quality of Life in Multiple Sclerosis

**DOI:** 10.3390/healthcare14142130

**Published:** 2026-07-16

**Authors:** Alexa M. Stuifbergen, Heather Becker

**Affiliations:** School of Nursing, The University of Texas at Austin, Austin, TX 78712, USA; hbecker@mail.nur.utexas.edu

**Keywords:** health promotion, quality of life, multiple sclerosis, health behaviors, longitudinal study, narrative review

## Abstract

**Background/Objectives**: In recent decades, health care for those living with chronic and disabling conditions has expanded beyond symptom and disease management to include efforts to promote health and quality of life. This narrative review synthesizes insights from a 26-year longitudinal study examining health promotion and quality of life among persons living with the chronic and disabling condition of multiple sclerosis (MS). **Methods**: The longitudinal study, launched in 1999, included a sample of 621 community-residing persons with MS. Our longitudinal research design allowed us to investigate unique issues related to the speed, sequence, direction, and duration of changes in a wide range of MS outcomes. Guided by Braun and Clarke’s thematic analysis framework, we have critically reviewed the more than 100 peer-reviewed publications and presentations generated by the study, in which we have identified overarching “Big Picture” themes, and we have contextualized them in relation to other MS research. **Results**: Five major themes were supported by the findings in this narrative review: (1) Strong interest in health promotion behaviors; (2) Positive view of overall health; (3) Mediation of the impact of functional limitations on quality of life; (4) Physical activity impacts the trajectory of functional limitations over time; and (5) Association of health behaviors with quality of life and health outcomes. **Conclusions**: Interventions that recognize and incorporate the positive health behaviors and perceptions of persons living with a long-term chronic condition can assist persons aging with chronic conditions to adjust to changes in their environment and the demands of self-management.

## 1. Introduction

An estimated 2.9 million people globally are living with multiple sclerosis (MS) [1]. MS is an unpredictable, chronic inflammatory demyelinating disorder in which immune-mediated damage to myelin disrupts electrical signal transmission in the central nervous system and can lead to axonal injury and loss. As a result, individuals with MS experience considerable variability in symptoms, impairments, and functional limitations. Diagnosed typically in early to mid-adulthood, often between the ages of 20 and 40, MS has a prolonged life-course impact on employment, family roles, and long-term health-related quality of life [2]. Although the disease management of MS has been significantly improved by biomedical, imaging, and pharmaceutical advances, individuals with this condition continue to face complex challenges that impact their overall health and well-being. Persons living with MS need guidance regarding their ability to sustain occupational and household responsibilities, the potential impact of MS on their future needs and life planning, and the role of their individual behaviors in shaping the trajectory of their disease-related limitations and disability and health outcomes [3].

For individuals with chronic conditions, the traditional focus of the health care system on symptom and disease management has expanded in recent decades to emphasize prevention, self-management, and the improvement in quality and years of life through a healthy lifestyle [4]. Health promotion refers to the process of enabling individuals to gain greater control over their health, thereby improving outcomes. It includes a broad range of activities designed to enhance well-being and support the attainment of optimal health across individuals, families, and communities [5]. In the scholarly literature and in healthcare practice and delivery, the severity of illness, functional limitations, and associated disabilities have received substantial attention as primary determinants of quality of life among persons with chronic disabling conditions. However, although this concern is justified, growing evidence suggests that the impact of illness may be influenced by mediating factors. Although progressive chronic conditions frequently follow a downward trajectory and are generally associated with increasingly adverse effects on quality of life over time, the development of key mediating factors—such as access to resources and active engagement in health-promoting behaviors—can help individuals sustain and occasionally enhance their quality of life [6,7].

For almost 30 years, we have collaborated to explore how health promotion strategies can enhance quality of life and health outcomes in individuals living with MS. During that time, we have witnessed the evolution of research about persons with MS from disease-oriented models to focus more broadly on health promotion and well-being. Many of these previous studies utilized cross-sectional designs investigating individual health promoting behaviors. By contrast, our recently completed 26-year longitudinal study of health promotion among persons with MS has a broader focus. Through longitudinal research focused on functional limitations, health promotion, and quality of life, we have sought to better understand the challenges encountered by individuals with MS and to identify factors that may mediate the effects of disease-related limitations on health and well-being. This body of work has enhanced knowledge of how people adjust and sustain life with chronic disabling conditions over extended periods [8]. While existing systematic reviews have focused on specific health behaviors among people with multiple sclerosis (MS), few have considered multiple domains of health behavior or their long-term associations with quality of life. To our knowledge, this is the first narrative review to synthesize more than two decades of evidence through a comprehensive health-promotion perspective, integrating the diverse behavioral, psychosocial, and lifestyle factors associated with quality of life in people with MS.

In this article, informed by Sukhera’s methods for narrative reviews [9], we synthesize and describe insights gleaned from our 26-year (1999–2024) study of health promotion and quality of life for persons living with MS. This longitudinal research, with repeated measurements over time, has enabled our examination of the pace, sequence, direction, and duration of change across a broad range of outcomes. Although our program of research has generated more than 100 peer-reviewed publications and presentations of individual studies and analyses, this synthesis of themes across this body of work as a “Big Picture” narrative will provide a more inclusive and generalized understanding of the impact of health promotion as a self-management strategy to support positive health outcomes for persons with MS.

## 2. Background

**Figure 1:**
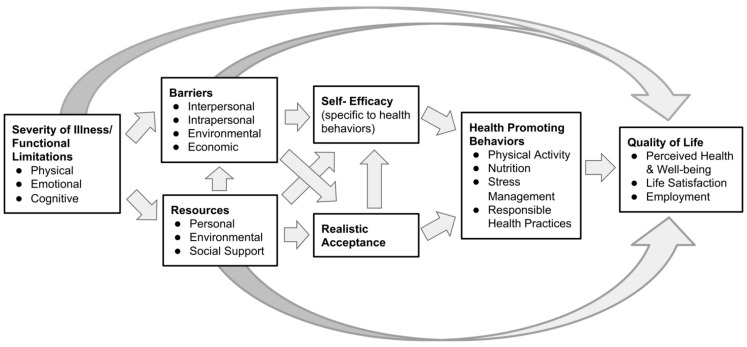
Refined model of health promotion and quality of life for persons with chronic conditions.

Both our longitudinal study and the present narrative review are based on a conceptual model of health promotion and quality of life informed by the existing literature and the qualitative and quantitative findings from our research with a sample of community-residing persons living with MS (Figure 1) [10,11,12,13]. Although other health promotion theoretical models exist, they do not explicitly address health promotion for people with chronic health conditions, such as MS [5]. We have used our data and that of other investigators to refine this conceptual model over time as an explanatory pathway for understanding the psychosocial and behavioral determinants of well-being in those with MS. For example, the resources construct (originally focused on social support) was expanded to include resilience as a personal resource. The model proposes that quality of life outcomes including perceived health and well-being as well as life satisfaction are the result of the direct and indirect influences of contextual, attitudinal, and behavioral factors (health-promoting behaviors). Statistical tests of the model with data from multiple samples [11,14] indicate that multiple dimensions of quality of life are influenced by the complex interplay of contextual factors (severity of illness and varied functional limitations), antecedent variables (perceived barriers, social support, self-efficacy, acceptance), and health-promoting behaviors. Health-promoting behaviors are conceptualized as a key mediator of the impact of functional limitations/severity of illness on quality-of-life outcomes. As disease severity fluctuates or increases over time, health-promoting behaviors can nurture an individual’s overall physical, mental, and social health as the individual achieves improvements in or maintenance of quality of life. The utility of this model has been demonstrated by its use to conceptually guide both descriptive and intervention research with multiple populations—cancer survivors, persons with post-polio syndrome, persons living with heart failure, persons with MS, women with HIV, women with fibromyalgia, and persons with diabetes [14,15,16,17,18,19,20,21].

## 3. Methodology

This narrative review was guided by Braun and Clarke’s (2006) six-phase framework for thematic analysis which includes: (1) familiarization with the data, (2) generation of initial codes, (3) searching for themes, (4) reviewing themes, (5) defining and naming themes, and (6) producing the report [22]. As emphasized by Braun and Clarke, thematic analysis is a flexible analytic method rather than a prescriptive or rigid methodology.

The review began with a comprehensive examination of all articles and abstracts generated from the longitudinal study, identified through the authors’ curricula vitae. Publications and presentations that were not data-driven or that reported findings from samples of individuals with MS outside the longitudinal cohort were excluded. Following this, meaningful features and distinctive findings from each included article and abstract were systematically identified. Subsequently, broader patterns across studies were examined, and conceptually related findings were organized into clusters. Through this iterative process, preliminary “big picture” themes were inductively developed to construct a coherent, meaning-centered interpretive account. These initial themes were then critically reviewed to ensure internal coherence within themes and clear distinctions between themes. The data set was revisited to confirm that the thematic structure adequately represented the full body of published and presented findings.

In the next phase, each “Big Picture” theme was refined, clearly defined, and appropriately named, with attention to its contribution to the overall interpretation of the findings [23]. Finally, the narrative synthesis was constructed by illustrating the themes and situating them within the broader literature by integrating findings from other investigators. Ongoing discussion, reflection, and refinement throughout this analytic process contributed to the development of the thematic structure presented in this review. The following paragraphs provide details of data collection from the longitudinal sample and interpretation of the themes.

### Data Collection

We began this study in 1999, in order to explore factors related to health promotion and quality of life among persons with MS [3]. A total of 749 individuals with MS who had taken part in a 1996 cross-sectional study and had consented to subsequent follow-up were mailed study information and consent materials. Among them, 621 respondents (83%) returned the survey and were enrolled in the longitudinal cohort. Participants have since received annual mailed surveys unless they requested withdrawal, died, became too ill to continue, or were lost to follow-up.

Over the course of the study, participation and return of surveys remained high, and the study’s basic questionnaire content and format remained consistent. In addition, a small number of unique items were added at the end of each questionnaire to address emerging areas of interest (e.g., cognitive issues, interest in biobanking, varied quality of life outcomes).

At the outset of the study in 1999, participants were between 21 and 81 years of age (mean 50.44, SD = 10.18). The group was predominantly female (83%), non-Hispanic white (93%), and married (73%). Most participants had at least a high school education (85%), and over one-third (35%) had completed college. Employment patterns showed that 25% were employed full-time, 32% were not working due to disability, and 13% were retired. Clinical characteristics indicated that 41% had relapsing–remitting MS, 18% primary progressive MS, 17% secondary progressive MS, and 11% progressive relapsing MS, while 14% either reported benign MS or were uncertain of their subtype. All individuals had been diagnosed for a minimum of 2 years, with an average disease duration of 13.5 years [3].

In 2024, 26 years after enrollment, 146 participants remained in the study. The year-26 sample was predominantly female (88.5%) and married (61.6%), with a mean age of 71.2 years (SD = 7.71); only 13.7% were still employed. Participants were generally well educated, with 45.4% holding a bachelor’s or graduate degree, and had been living with their diagnosis for an average of 36.2 years (SD = 5.7). Starting in 2002 (Year 4), we began recording specific causes for participant status changes among individuals no longer retained in the study. Death accounted for the largest proportion of attrition (*n* = 153), and 85 participants were deemed lost to follow-up when mailed surveys were returned and could not be re-delivered or traced using available contact details; some of these cases may also reflect unreported deaths. Over the 26 years of follow-up, only 55 individuals from the initial sample of 621 asked to discontinue participation [8].

## 4. Thematic Findings

### 4.1. Strong Interest in Health-Promoting Behaviors

One of the most salient findings of this longitudinal study was the participants’ sustained interest in and perceived value of health-promoting behaviors, reflected in consistently high survey response rates over time, ranging from 88.6% in Year 3 to 65.7% in Year 25 [8]. Qualitative feedback further underscored the personal and communal significance of the research, with participants frequently adding spontaneous written comments to explain their responses or to contextualize their experiences (e.g., changes in health status, loss of a spouse, or comorbid health conditions) and responding to a final open-ended question asking if there was anything else they wished to add. Completing the annual survey required substantial effort for many participants because of fatigue or fine motor difficulties, yet engagement remained high. Several participants described survey completion as an intervention in itself; it served as a reminder of recommended health behaviors and reinforced self-reflection and motivation. Expressions such as “So glad someone is finally studying what works for us” and “My participation has made me feel valued and an important part in helping others” illustrate the depth of participants’ commitment and their perceived relevance of health promotion in their lives [23,24]. Other investigators in a variety of countries (Australia, Germany, England) have reported that persons with MS have strong interest, with high value, engagement, and participation in both in-person and digital interventions focused on health promoting behaviors and lifestyle management [16,25,26].

### 4.2. Positive Views of Overall Health

Self-rated health—an individual’s subjective, single-item assessment of overall health—has consistently been shown to be a powerful predictor of morbidity and mortality across diverse populations [27]. In each annual survey, participants were asked to rate their overall health as poor, fair, good, or excellent. Despite living with a chronic disabling condition characterized by an uncertain prognosis and a variable disease trajectory, the majority of participants viewed their overall health as good or excellent at the initiation of the study and across time [28,29]. Particularly striking is that after more than 25 years of living with MS, only 3% rated their health as “poor”; 60% rated their overall health as good or excellent. In addition to their positive ratings of overall health, participants consistently endorsed a functional and wellness-oriented definition of health, emphasizing their ability to do the things they wanted and needed to do—rather than suggesting biomedical models focused primarily on diagnosis, medical treatment, or disease severity.

This theme is consistent with the literature supporting multidimensional views of health [30,31], including among those living with a chronic condition or disability. Krahn et al. [30] have proposed that health is distinct from illness and function and that good health can thus exist in the presence of limitations associated with chronic illness, disability, or aging. Their proposed definition of health acknowledges the contextualization of individual meanings of health in life experiences and highlights the importance of environmental and social factors on health.

### 4.3. Mediation of the Impact of Functional Limitations on Quality of Life

Although greater functional limitations were negatively associated with quality of life, this relationship was consistently mediated by psychosocial and behavioral factors, including perceived barriers, self-efficacy, social support, acceptance, and engagement in health-promoting behaviors [3,11,32]. Barriers to health promotion such as fatigue, mobility limitations, and lack of access were consistently associated with lower social support, less frequent engagement in health behaviors, and greater functional limitations and higher depressive symptoms [33,34]. Fatigue (feeling too tired) was the highest ranked barrier at all time points, but the relative importance of other perceived barriers shifted over time, reflecting changes in life circumstances, adaptation, and disease progression.

Consistent with a substantial body of prior research, self-efficacy emerged as a central predictor of health-promoting behaviors [7,35]. Individuals who have confidence in their ability to manage their health are more likely to engage in behaviors that support physical and psychosocial well-being, thereby enhancing their quality of life. Psychosocial resources, including social support and acceptance, also played a protective role, both mediating the impact of functional limitations and demonstrating positive direct associations with quality-of-life outcomes. Collectively, these factors buffered the negative effects of disease-related impairment, supporting resilience, well-being, and sustained quality of life over time [32,36].

While many research teams have explored predictive variables of change in quality of life in longitudinal studies ranging from 12 months to 10 years [6,7,37,38,39], few have specifically addressed the mediation of the impact of functional limitations on quality of life. Using hierarchical linear model analyses, Wolin et al. [7] reported that adding stress, self-efficacy, and social support measures significantly improved their model’s prediction of quality of life after controlling for disease severity/duration. Koelmel et al. [39] found that resilience mediated the relationship between various social supports and mental health outcomes.

### 4.4. Physical Activity Impacts the Trajectory of Functional Limitations

Data from our longitudinal study demonstrate that the progression of functional limitations is associated with engagement in health-promoting behaviors, with physical activity exerting the strongest effect over time. Multivariate latent curve modeling was used to estimate changes in functional limitations at both the population and individual levels. This analytic approach is particularly well suited to the study of MS, because it accommodates individual variability in rates of change over time—an essential consideration, given the heterogenous, fluctuating course of the disease. In addition, these techniques permit the simultaneous examination of multiple outcomes and correlations among their trajectories of change.

Across analyses with 7 years of follow-ups and 11 years of follow-ups, rates of change in functional limitations were negatively correlated with rates of engagement in physical activity behaviors and with quality-of-life ratings. Thus, higher levels of physical activity were associated with slower progression of functional limitations and better quality of life over time. Furthermore, physical activity levels measured at Time 1 were inversely associated with subsequent changes in functional limitations, suggesting that individuals who were more active at baseline tended to experience fewer or decreasing limitations over time [3,40].

Several other investigators have confirmed similar findings in cross-sectional or longitudinal studies of physical activity and MS progression. In a cross-sectional study, Gromish et al. [41] found that physical activity was the only lifestyle behavior associated with functional capabilities. Hedström et al. [42] analyzed data from 3284 individuals with relapsing-remitting MS who were followed for up to 15 years through the Swedish MS Registry. Their findings were similar to ours: higher levels of physical activity at diagnosis were associated with reduced risk of disability progression, and in a subsample (*n* = 1724) with measurement of post-diagnosis physical activity levels, increased activity was associated with more favorable disability-related outcomes. Guo et al. [43] recently applied latent class trajectory modeling to EDSS data from 3163 individuals newly diagnosed with relapsing-remitting MS and followed through the Swedish Epidemiological Investigation of MS and identified seven distinct trajectories of disability progression. High physical activity decreased the odds of increasing disability.

### 4.5. Association of Health Behaviors with Quality of Life and Health Outcomes

Across multiple time points, greater engagement in health-promoting behaviors was consistently associated with higher quality of life outcomes. The frequency of health behaviors was moderately correlated (>0.40) with various indicators of quality of life [44]. Similarly, Zhang et al. [45] found that SF-36 Role Physical and Social Functioning subscale scores (quality of life indicators) were moderately correlated with the frequency of health behaviors in those with both progressive and non-progressive forms of MS. Extending these findings, analyses of 5 years of data spanning the period before and during the COVID-10 pandemic showed that both health-promoting behaviors and quality of life remained stable over time, suggesting a potential protective effect of sustained engagement in these behaviors during periods of widespread disruption [46].

There was very limited investigation of health promotion and quality of life among persons with MS during the early years of this longitudinal study. However, in more recent years, there have been a large number of reports linking specific health behaviors (e.g., physical activity, diet, positive attitude) to quality of life in cross-sectional and intervention studies [47,48,49]. More recent longitudinal analyses of the relationship of health behaviors and changes in quality of life have generally focused on a single health behavior and explored changes over shorter time periods. In a 3-month follow-up to a dietary intervention with 50 persons with MS, Hedfi et al. [50] found that greater adherence to a Mediterranean diet was associated with significant improvement in quality-of-life scores, especially in mental health domains. Similarly, Yu et al. [51] found that persons with MS in the UK MS Register data (*N* = 817) who reported greater use of an alternate Mediterranean diet had better quality of life outcomes at a 6-year follow up, although these benefits were limited to females. Healy et al. [52] explored the association between physical activity and health-related quality of life (HRQOL) over a 2-year period. This study specifically addressed variations in physical activity from leisure activity to moderate and strenuous activity. Higher levels of leisure activity were associated with better HRQOL outcomes, and increases in mild physical activity over 2 years were moderately associated with improvement on the measure of quality of Ife.

Fidao et al. [53] provided one of the few investigations of the impact of multiple health behaviors on quality of life among participants in the online HOLISM study in Australia. Their analysis included the 602 participants who completed assessments at baseline and at 2.5 years, 5 years, and 7.5 years follow up. While the single behaviors of diet and physical activity contributed significantly to better quality of life, engagement with multiple lifestyle behaviors (diet, meditation, physical activity, smoking cessation, vitamin D) provided additional benefits. Prospectively, engagement with three or more behaviors was associated with both mental and physical quality of life and the strongest associations were found for engagement in five behaviors.

## 5. Discussion

This narrative review synthesizes findings from a 26-year longitudinal program of research examining health promotion and quality of life among people living with MS. Together, with results reported by other researchers, these findings provide evidence that individuals can maintain—and in some cases enhance—quality of life while aging with a chronic, progressive neurological condition. Rather than merely adapting or coping, many participants demonstrated the capacity to thrive within the context of illness, challenging common deficit-based interpretations of chronic disease trajectories.

One of the most important insights emerging from this long-term program of research is the value of integrating both quantitative and qualitative approaches to fully understand the lived experience of those with MS and the role of health promotion over time. The primarily quantitative analyses documented robust and consistent relationships among functional limitations, health-promoting behaviors, and multiple dimensions of quality of life. At the same time, additional qualitative feedback offered critical contextual depth, revealing how participants made meaning of their illness, navigated changing life circumstances, and sustained motivation to engage in health-promoting behaviors despite increasing functional challenges. These complementary approaches illuminate not only what changes over time, but how and why individuals persist in self-management and wellness-oriented behaviors across decades of illness. Similarly, von Glasenapp et al. [25] reported the use of mixed methods as a valuable approach to better understand the factors influencing health behavior changes in persons with MS (*N* = 234) participating in the POWER@MS1 trial in Germany. Qualitative interviews with health care providers and persons with MS elucidated aspects of the intervention viewed as most helpful as well as the desire for personal consultation to supplement a personalized digital lifestyle management application.

Longitudinal studies that permit examination of both group-level trends and individual-level variability are particularly well suited for conditions such as MS, lupus, arthritis, and other chronic illnesses characterized by heterogeneous trajectories of progression and symptom fluctuation. The use of repeated measures over extended periods enabled the identification of patterns that would not be detectable in cross-sectional or short-term studies—such as the stability of health-promoting behaviors, the relative slowing of functional decline associated with higher physical activity, and the enduring influence of psychosocial resources. Equally important, analytic approaches that accommodate individual differences in rates and directions of change show that illness trajectories are not uniform and that meaningful variation exists in how people adapt and respond over time. Longitudinal analyses that rely solely on change at the group level (means or medians) may miss the subtle individual functional changes that often occur in persons with MS.

In our early conceptualizations that guided this research, we assumed that health-promoting behaviors would primarily mediate the impact of impairment and functional limitations on quality of life. Consistent with this view, behaviors such as regular physical activity were understood as mechanisms to reduce deconditioning and preserve function, without necessarily altering disease pathology. Over time, however, both quantitative findings and emerging evidence from related fields suggested a more expansive role for health promotion. Engagement in behaviors such as physical activity, cognitive stimulation, and potentially dietary practices are not only associated with the trajectory of functional limitations but intervention work of other investigators suggests that these behaviors may also contribute to changes in disease processes themselves, including inflammation and cognitive functioning [54,55]. A recent integrative synthesis by Lanzillo and colleagues reviewed evidence linking modifiable lifestyle factors with MS risk, progression and clinical outcomes [56]. Their findings support a biologically plausible and clinically relevant contribution for lifestyle factors in immune regulation, metabolic balance, vascular function, and neuroinflammatory processes. Although this area remains a “work in progress,” these findings support a more dynamic, potentially transformative understanding of health promotion in chronic illness and argue for more rigorously designed methodologies to investigate this potential.

Findings from this long-term study also challenge the assumption that relationships among illness severity, health-promoting behaviors, and quality of life are unidirectional. Instead, evidence increasingly supports a bidirectional or recursive model in which health-promoting behaviors both influence and are influenced by functional status, psychosocial resources, and quality of life. For example, higher engagement in physical activity was associated with slower progression of functional limitations, while preserved function and positive health perceptions likely reinforced continued engagement in health-promoting behaviors. The extended timeframe of this study was essential in revealing these reciprocal relationships, and it underscores the value of long-term longitudinal research for theory refinement.

An important gap in this body of research is the limited examination of broader policy and structural influences on health promotion and quality of life. Employment policies, disability accommodations, access to person-centered healthcare, and national differences in social support systems likely exert substantial effects on individuals’ capacity to engage in health-promoting behaviors. Policies such as the Americans with Disabilities Act and workplace flexibility may play a critical role in sustaining employment, social participation, and overall quality of life, yet we did not explicitly examine those factors in this study. Future research should more fully integrate policy contexts, particularly in cross-national or comparative studies.

Despite its strengths, this longitudinal program of research has several limitations that warrant consideration. As the investigators for this longitudinal study, our thematic analysis may be influenced by author bias. Other investigators may provide evidence challenging the utility and validity of these themes in the future. Our narrative review was limited to the findings of a longitudinal investigation of health promotion and quality of life in a single well-educated sample. The sample represents a convenience cohort of individuals from one large geographic region of the U.S. who were willing to participate in long-term research and who might have been more engaged in health promotion than the broader MS population. Consequently, there is limited understanding of individuals with fewer social and economic resources, as well as those who are less engaged in self-management or health promotion activities, including non-responders and participants lost to follow-up for reasons other than mortality.

The extended follow-up period of 26 years magnified a common challenge inherent in longitudinal research—participant attrition and the potential bias it introduces. Although response rates among actively enrolled participants remained consistently high over time, many original participants were lost due to death, increasing impairment that prevented continued participation, or the research team’s inability to maintain updated contact information. In fact, “dropouts” were significantly older and had greater functional limitations at the initiation of the study, than those who continued to participate [3]. Consequently, many publications based on this longitudinal study reflect a sample of “survivors,” which differs from the broader population of individuals with MS who were initially enrolled but no longer participated [3,8,29,40]. It is likely that those who remained in the study were more engaged with their health and better able—due to available resources, fewer limitations, or greater support—to sustain participation over such an extended period. As a result, this subgroup may not fully represent the wider population of persons with MS.

Additional gaps include the absence of individuals newly diagnosed with MS and the limited examination of how engagement in health promotion varies with stage of illness or life course. Attrition due to mortality, while expected in a long-term study of aging with MS, further complicates interpretation of long-term trends. Moreover, we did not directly assess health literacy, disease-related knowledge, or digital literacy—factors that are increasingly important as healthcare and self-management resources shift to digital platforms. Understanding how digital access and skills shape engagement in health promotion represents a critical area for future research, particularly for persons with chronic and disabling conditions. For example, future studies could explore whether lower digital literacy predicts reduced engagement in telehealth-based wellness interventions or outcomes and whether this relationship varies by age, education, socioeconomic status, disability level, or disease severity.

## 6. Conclusions

This 26-year longitudinal program of research demonstrates that health-promoting behaviors play a central role in supporting quality of life among individuals living with MS, even in the presence of progressive functional limitations. Our evidence is consistent with that of other researchers and clearly shows that health-promoting behaviors are meaningfully related to functional outcomes, psychosocial well-being, and perceived health. Our findings also support the importance of conceptualizing health promotion as a dynamic, reciprocal process with the potential to alter trajectories of functioning and well-being. Future research should build on these insights by incorporating more diverse samples, examining policy and digital contexts, and continuing to refine models that reflect the complex, iterative nature of the impact of health-promoting behaviors for persons with chronic conditions.

Collectively, this program of research contributes actionable insights for clinicians, rehabilitation specialists, and public health professionals. Interventions that build self-efficacy, reduce perceived barriers, and strengthen psychosocial resources including social support and acceptance have the potential to substantially improve the lived experiences of individuals with MS. These findings support a shift away from deficit-oriented models of care toward strengths-based approaches that recognize individuals as active agents in shaping their health and well-being across the illness trajectory. Low-cost, accessible health promotion strategies are particularly promising as complementary approaches to biomedical treatment, with relevance for diverse clinical and community settings.

## Data Availability

No new data were created or analyzed in this study.
